# *Bacillus subtilis* QST 713 Supplementation during Late Gestation in Gilts Reduces Stillbirth and Increases Piglet Birth Weight

**DOI:** 10.1155/2022/2462241

**Published:** 2022-06-06

**Authors:** Nguyen Hoai Nam, Nguyen Duc Truong, Dao Thi Ha Thanh, Pham Ngoc Duan, Tran Minh Hai, Bui Tran Anh Dao, Peerapol Sukon

**Affiliations:** ^1^Department of Animal Surgery and Theriogenology, Faculty of Veterinary Medicine, Vietnam National University of Agriculture, Hanoi, Vietnam; ^2^Department of Parasitology, National Institute of Veterinary Research, Hanoi, Vietnam; ^3^Department of Parasitology, Hanoi Medical University, Hanoi, Vietnam; ^4^Department of Pathology, Faculty of Veterinary Medicine, Vietnam National University of Agriculture, Hanoi, Vietnam; ^5^Division of Anatomy, Faculty of Veterinary Medicine, Khon Kaen University, Khon Kaen, Thailand; ^6^Research Program on Toxic Substances, Microorganisms and Feed Additives in Livestock and Aquatic Animals for Food Safety, Khon Kaen University, Khon Kaen, Thailand

## Abstract

Recent studies have shown that probiotic supplementation during late gestation exerts some beneficial effects on reproductive performance of the sows. This study aimed to investigate effects of *Bacillus subtilis* QST 713 supplementation in gilts on different reproductive criteria. A total of 94 Camborough-48 gilts at day 85 of gestation were randomly allocated into 2 groups: (1) control diet; (2) control diet + 4 × 10^8^ CFU *Bacillus subtilis* QST 713 per day. Gilts were supplemented until farrowing. At farrowing, litter size, number of piglets born alive, stillbirths, mummies, birth weight, farrowing duration, and birth interval were recorded. Within litter variation of piglet birth weight, depicted as SDBW and CVBW, was also calculated. Results showed that *Bacillus subtilis* QST 713 supplementation decreased stillbirth rate (1.26 vs. 4.37%, *p*=0.035) and increased birth weight of the piglets (1303.94 vs. 1234.09 g, *p*=0.007). Also, the litter size (11.85 vs. 10.67, *p*=0.03), number of piglets born alive (11.71 vs. 10.23, *p*=0.008), and litter weight (15473.06 vs. 13174.86 g, *p*=0.002) in the treatment group were higher than those in the control. Farrowing duration (174.39 vs. 160.81 minutes, *p*=0.162), birth interval (16.32 vs. 16.59 minutes, *p*=0.674), SDBW (85.07 vs. 94.65 g, *p*=0.343), and CVBW (6.42 vs. 7.85, *p*=0.12) were independent of the *Bacillus subtilis* QST 713 supplementation. Results of the present study indicate that supplementation of *Bacillus subtilis* QST 713 during late gestation in gilts reduces stillbirth and increases birth weight thereby improving their reproductive performance.

## 1. Introduction

During late gestation, the richness of microbiota in intestine of the sows reduced [1, 2] while fecal endotoxin increased [1]. Besides, an increase in gut permeability results in increased plasma endotoxin concentration in sows [1]. These changes may influence sows' digestion, absorption, and nutrient metabolism which may affect the birth weight of piglets [3].

Recently, there is an increase in studies investigating effects of probiotic supplementation at late gestation on reproductive performance of sows [4–8]. Some researchers have shown that sows supplemented with probiotic had larger litter sizes [5, 8], higher numbers of piglets born alive [5, 8, 9], and heavier litter weights [7, 8, 10]. Zhang et al. [5] also found that probiotic supplementation could reduce farrowing duration and birth interval.

*Bacillus subtilis* QST 713 used in this study was isolated from soil in California, USA, in 1995. This strain is reported to be low toxic to mammals and not likely to be a pathogenic agent in human. Supplementation of *Bacillus subtilis* QST 713 was suggested to be an effective approach to control necrotic enteritis in broilers [11]. Furthermore, *Bacillus subtilis* QST 713 increased the length of intestinal villi and decreased the number of deep crypts [12]. *Bacillus subtilis* QST 713 supplementation also suppressed the harmful effect of antimicrobial on beneficial bacteria in the intestine of the weaned pigs thereby working as “a preventative and/or attenuating compound against dysbiosis in piglets” [13]. All of those findings suggest that *Bacillus subtilis* QST 713 may increase feed digestion and nutrients absorption in animals. Based on available reports in beneficial effects of *Bacillus subtilis* QST 713 in animals, we hypothesized that this strain might improve reproductive performance of the pig. The reason to this hypothesis was that the increased nutrients absorption might lead to the increased birth weight and subsequently decreased stillbirth rate in newborn piglets. To this end, the present study aimed to evaluate effects of *Bacillus subtilis* QST 713 supplementation from day 85 of gestation to farrowing on different reproductive criteria including the number of piglets born alive, litter birth weight, stillbirth rate, dead born rate, birth weight, within litter variation of piglet birth weight, farrowing duration, and birth interval.

## 2. Materials and Methods

### 2.1. Animal Care

The present experiment was reviewed and approved by the Committee on Animal Research and Ethics of Faculty of Veterinary Medicine, Vietnam National University of Agriculture (CARE-2021/01).

### 2.2. Animals, Diet, and Experimental Design

This study was conducted in a commercial pig farm during February and July, 2021 in Bavi, Hanoi, Vietnam, enrolling ninety-four Camborough-48 gilts. Young gilts were vaccinated against porcine reproductive and respiratory syndrome, Aujeszky's disease, classical swine fever, foot and mouth disease, and porcine circovirus during 24–29 weeks of age. At the age of 30 weeks, estrus gilts were bred with diluted semen of PIC®337 boars. Pregnant gilts were vaccinated against classical swine fever, foot and mouth disease, and *Escherichia coli*. During the first 84 days of gestation, gilts were fed 1.8–2 kg of a gestation feed (GF07, Greendfeed Vienam). From days 85 to 110, gilts were fed 2.0–2.2 kg of a lactation feed (GF08, Greenfeed, Vietnam). Between day 111 and farrowing gilts received 1.0–1.5 kg of the lactation feed. The nutrient composition of the gestation and lactation feed was shown in Table 1. At day 85 of gestation, 94 gilts were randomly assigned into 2 groups with 47 each. In the treatment group, gilts were daily supplemented with 4 × 10^8^ CFU *Bacillus subtilis* QST 713 (Baymix, GROBIG® BS, Bayer de Mexico, S.A. de C.V., Mexico) in 4 g mixture of the probiotic and dextrose, which was topped to the daily feed (GF08, Greedfeed, Vietnam). Gilts in the control group received 4 g dextrose. Pregnant gilts were individually housed in gestation crates, and moved to farrowing crates at about day 107 of gestation. All animals were *ad libitum* accessed to water provided through a bite nipple drinking system.

### 2.3. Data Collection

At parturition, gilts were supervised by two trained veterinarians. Litter size (total born), number of born alive piglets, dead born piglets (stillbirth and mummy), gestation length, birth interval, farrowing duration, and birth weight were recorded. Mummified piglets were those born dead with full brown/black color of skin. Other born dead piglets were classified as stillbirth. Stillborn piglets included intrapartum and prepartum stillbirths. However, postmortem examination was not allowed due to the regulation of the farm then these two types of stillbirth were not distinguished. Birth interval was the time period between the births of two consecutive piglets. Farrowing duration was the interval between the birth of the first piglet and the birth of the last piglet. Piglets were individually weighed with a 5 g precision digital scale before colostrum intake. Mean birth weight (MBW) of each litter was calculated by dividing litter weight by litter size. Standard deviation of mean birth weight of each liter (SDBW) was also calculated. Coefficient of variation of birth weight of each litter (CVBW) was derived by dividing SDBM by MBW then multiplying with 100.

### 2.4. Statistical Analysis

To deal with hierarchical data where multiple piglets born from a sow, generalized linear mixed model was used to compare the stillbirth rate and dead born rate of piglets in the treatment and control groups. Similarly, linear mixed effect model was used to compare piglet birth weight and birth interval between treatment and control groups. Litter size, number of piglets born alive, litter weight, SDBW, CVBW, and farrowing duration were compared between treatment and control groups using a *t*-test. All tests were conducted in RStudio Desktop 1.3.1093 (Boston, MA, RStudio Team: Integrated Development for *R*). A *p* value <0.05 was considered significant.

## 3. Results

Among 94 gilts enrolled in the study, 10 gilts farrowed in late evening, so were not supervised and discarded from the study. Among 84 gilts remained, 41 gilts were in the treatment and 43 gilts were in the control groups. In total, 939 piglets were born from 84 gilts. Among them 910 (96.91%) piglets were born alive, 26 (2.77%) were stillborn, and 3 (0.32%) were born as mummies. The overall incidence of stillbirth at farrowing level was 21.43% (18/84), and the birth weight was 1269.76 ± 154.09 g. The farrowing duration and birth interval were 167.44 ± 44.38, and 16.45 ± 9.14 minutes, respectively.

*Bacillus subtilis* QST 713 supplementation did not influence gestation length (116.12 vs. 116.56 days), farrowing duration (174.39 vs. 160.81 minutes), birth interval (16.32 vs. 16.59 minutes), incidence of stillbirth at litter level (14.63 vs. 27.91%), SDBW (85.07 vs. 94.65 g), and CVBW (6.42 vs. 7.85%). *Bacillus subtilis* QST 713 supplementation increased litter size (11.85 vs. 10.67, *p*=0.03), number of piglets born alive (11.71 vs. 10.23, *p*=0.008), and the litter weight (15473.06 vs. 13174.86, *p*=0.002). Supplementation of *Bacillus subtilis* QST 713 decreased stillbirth rate (1.26 vs. 4.37%, *p*=0.035) and dead born rate (1.46 vs. 4.78, *p*=0.028), and increased birth weight of piglets (1303.94 vs. 1234.09, *p*=0.007) (Table 2).

## 4. Discussion

The present study revealed that *Bacillus subtilis* QST 713 supplementation both decreased the stillbirth rate and increased the birth weight. Interestingly, the increased birth weight was simultaneously present with a higher litter size and higher number of piglets born alive. Moreover, despite the larger litter size, the within litter variation of piglet birth weight depicted as SDBW and CVBW in the treatment group was numerically lower than that in the control group.

The larger litter size in the treatment group in this study reflected the results of some previous studies [5, 8]. Other studies also reported a numerically higher litter size in probiotic supplementation groups in comparison with that in control groups [6, 7]. However, as discussed by Zhang et al. [5] the increased litter size in the treatment group is independent of probiotic supplementation because the litter size had already been determined before the use of probiotics. By contrast, the number of piglets born alive (NBA) which is the result of total born minus the number of piglets born dead (NBD) might be influenced by the probiotic supplementation. The increased NBA in the treatment group was in agreement with some previous findings [5, 8, 9]. However, many other studies reported nonsignificant effect of probiotic supplementation on this criterion [4, 6, 7, 10, 14–20]. The positive effect of the probiotic supplementation on NBA may be partly attributable to the fact that it reduced stillbirth rate. Previous studies demonstrated that probiotic supplementation did not reduce piglet stillbirth rate [4–9]. It has been shown that stillbirth decreased when birth weight increased [21–24]. Therefore, the reduced stillbirth rate in the treatment group may be due to the increased birth weight (1303.9 ± 161.5 g vs. 1234.1 ± 137.3 g).

It is interesting that *Bacillus subtilis* QST 713 increased both litter size/NBA and birth weight despite the fact that birth weight is negatively associated with litter size [25, 26]. Some previous studies failed to find any beneficial effects of probiotic supplementation at late gestation on birth weight of piglets [7–9, 20] while other studies even found a reduced birth weight in the probiotic supplementation group where litter and/or NBA increased in comparison with that in the control [5, 14]. Two previous studies found that birth weight was increased while litter sizes were unaltered when probiotic was supplemented throughout 2 successive estrus cycles [27] or late gestation [28]. However, in the former study, the positive effect of probiotic on birth weight only exhibited in the second estrus cycle [27], and in the latter one, the increased birth weight seemed to be attributable to isomaltooligosaccharide rather than probiotic [28].

It is well documented that the litter size positively correlates with within litter variation of piglet birth weight [29–31]. In other words, a larger litter size results in a larger variation of piglet birth weight. However, in the present study, the probiotic supplementation increased litter size while did not increase within litter variation of piglet birth weight. This finding can be partly explained via the increased birth weight in the treatment group because birth weight has been found negatively associated with CVBW [29].

In the present study, the effect of *Bacillus subtilis* QST 713 supplementation on farrowing duration and birth interval is diverse from the finding of the solely existed study that evaluated this aspect [5]. In their study Zhang et al. [5] speculated that the sows and piglets in the treatment group might be more physically stronger, therefore, the farrowing duration and birth interval were reduced. It is well established that litter sizes are positively associated with farrowing duration [26, 32], and birth weight positively correlated with birth interval [25]. Therefore, the increased litter size and birth weight might mask any potentially beneficial effect of *Bacillus subtilis* QST 713 supplementation on farrowing duration and birth interval resulting in nonsignificant difference in the present study.

The mechanism of action of *Bacillus subtilis* QST 713 supplementation on gilt reproductive performance is not totally clear. Previous studies showed that *Bacillus subtilis* improve the intestinal immune status [33], and immunological response to vaccination [34, 35]. *Bacillus* spp. can produce a wide range of antimicrobial substances which were found to inbibit the growth of many pathogenic bacteria such as *Clostridium difficile*, *Campylobacter jejuni*, *Streptococcus pneumoniae*, *Campylobacter coli*, *Proteus vulgaris*, *Klebsiella pneumoniae*, *Pseudomonas aeruginosa*, and *Salmonella typhi* [36, 37]. These inhibitions might lead to lowered sera endotoxin concentration in sows supplemented with *Bacillus subtilis* during the late stage of gestation [5]. Tactacan et al. [11] reported that *Bacillus subtilis* QST 713 could also produce some antimicrobials although the names of these products were not specified. In chicken, *Bacillus subtilis* QST 713 stimulated the development of intestinal mucosa and villi, and the growth of *Lactobacillus* spp., and inhibited *Escherichia coli* and *Enterococcus* spp. [12]. In the pig, *Bacillus subtilis* QST 713 prevented the growth of *Escherichia coli*, and promoted the development of beneficial bacteria such as *Bulleidia* [13]. The beneficial effects of *Bacillus* spp., *Bacillus subtilis*, and *Bacillus subtilis* QST 713 lead to the suggestion that supplementation of *Bacillus subtilis* QST 713 during the late gestation might enhance the intestinal immune status and the health of gut microbiota, increase the digestion and absorption of nutrients in treated gilts. These promoting effects resulted in enhanced nutrient delivery to the swine fetuses which subsequently increased birth weight, and decreased stillbirth rate.

The present study had a limitation. We used commercially industrialized diets in which only some main ingredients are listed without any further information. This led to the difficulty of formulation of such diets in the future studies. Nevertheless, the promising beneficial effect of *Bacillus subtilis* QST 713 on reproductive performance of gilts/sows deserves further investigation in the future.

## 5. Conclusion and Recommendations

The present study suggested that supplementation of *Bacillus subtilis* QST 713 at the dose of 4 × 10^8^ cfu/meal/day from day 85 of gestation to farrowing in gilts could decrease stillbirth rate and increase the birth weight. The increase birth weight and decreased stillbirth rate were simultaneously present with a larger litter size/NBA. *Bacillus subtilis* QST 713 is a promising probiotic supplement in gilts during late gestation for improvement of reproductive performance.

## Figures and Tables

**Table 1:** Nutrient composition of basal diets.

Nutrient composition	Gestation diet	Lactation diet
Metabolizable (kcal/kg)	3000	3200
Minimum crude protein (%)	14	16.50
Maximum crude fiber (%)	10	6
Calcium (%)	0.9–1.5	0.9–1.5
Phosphorus (%)	0.6–1.2	0.6–1.2
Total lysine (%)	0.80	0.95
Methionine + cystein (%)	0.50	0.55

Ingredients of gestation and lactation diets: soybean meal, animal protein, rice, rice bran, corn, wheat bran, casava root, amino acids, vitamins, and minerals. Gestation diet was used during the first 84 days of gestation. Lactation diet was used from day 85 of gestation to farrowing.

**Table 2:** Reproductive performance of the gilts at farrowing.

Parameters	Treatment	Control	*p* value
Litter size	11.85 ± 2.19	10.67 ± 2.70	0.030
Number born alive	11.71 ± 2.18	10.23 ± 2.78	0.008
Stillbirth rate (%)	1.26 (6/478)	4.37 (20/458)	0.035
Dead born rate (%)^*∗*^	1.46 (7/479)	4.78 (22/460)	0.028
Incidence of stillbirth at litter level (%)	14.63 (6/41)	27.91 (12/43)	0.138
Gestation length (day)	116.12 ± 1.45	116.56 ± 1.67	0.205
Birth weight (g)	1303.94 ± 161.54	1234.09 ± 137.27	0.007
Litter weight (g)	15473.06 ± 3090.63	13174.86 ± 3332.76	0.002
SDBW (g)	85.07 ± 39.61	94.65 ± 51.86	0.343
CVBW (%)	6.42 ± 2.61	7.85 ± 5.32	0.120
Birth interval (min)	16.32 ± 9.18	16.59 ± 9.12	0.674
Farrowing duration (min)	174.39 ± 43.34	160.81 ± 44.85	0.162

The results are presented as mean ± standard deviation; *p*: probability. Litter size included live born, stillborn, and mummified piglets. Dead born piglets included stillborn and mummified piglets. SDBW: standard deviation of mean birth weight of litter. CVBW: coefficient of variation of mean birth weight of litter.

## Data Availability

The data can be acquired from the principal author upon reasonable request.

## References

[1] Cheng C., Wei H., Yu H., Xu C., Jiang S., Peng J. (2018). Metabolic syndrome during perinatal period in sows and the link with gut microbiota and metabolites. *Frontiers in Microbiology*.

[2] Kong X. F., Ji Y. J., Li H. W. (2016). Colonic luminal microbiota and bacterial metabolite composition in pregnant Huanjiang mini-pigs: effects of food composition at different times of pregnancy. *Scientific Reports*.

[3] Kranendonk G., Van der Mheen H., Fillerup M., Hopster H. (2007). Social rank of pregnant sows affects their body weight gain and behavior and performance of the offspring1, 2. *Journal of Animal Science*.

[4] Hu J., Kim Y. H., Kim I. H. (2021). Effects of two bacillus strains probiotic supplement on reproduction performance, nutrient digestibility, blood profile, fecal score, excreta odor contents and fecal microflora in lactation sows, and growth performance in sucking piglets. *Livestock Science*.

[5] Zhang Q., Li J., Cao M. (2020). Dietary supplementation of *Bacillus subtilis* PB6 improves sow reproductive performance and reduces piglet birth intervals. *Animal Nutrition*.

[6] Menegat M. B., DeRouchey J. M., Woodworth J. C., Dritz S. S., Tokach M. D., Goodband R. D. (2019). Effects of *Bacillus subtilis* C-3102 on sow and progeny performance, fecal consistency, and fecal microbes during gestation, lactation, and nursery periods1, 2. *Journal of Animal Science*.

[7] Hayakawa T., Masuda T., Kurosawa D., Tsukahara T. (2016). Dietary administration of probiotics to sows and/or their neonates improves the reproductive performance, incidence of post-weaning diarrhea and histopathological parameters in the intestine of weaned piglets. *Animal Science Journal*.

[8] Baker A. A., Davis E., Spencer J. D., Moser R., Rehberger T. (2013). The effect of a *Bacillus*-based direct-fed microbial supplemented to sows on the gastrointestinal microbiota of their neonatal piglets. *Journal of Animal Science*.

[9] Böhmer B. M., Kramer W., Roth-Maier D. A. (2006). Dietary probiotic supplementation and resulting effects on performance, health status, and microbial characteristics of primiparous sows. *Journal of Animal Physiology and Animal Nutrition*.

[10] Wang J., Ji H. F., Hou C. L. (2014). Effects of *Lactobacillus johnsonii* XS4 supplementation on reproductive performance, gut environment, and blood biochemical and immunological index in lactating sows. *Livestock Science*.

[11] Tactacan G. B., Schmidt J. K., Miille M., Jimenez D. (2013). A *Bacillus subtilis* (QST 713) spore-based probiotic for necrotic enteritis control in broiler chickens. *The Journal of Applied Poultry Research*.

[12] Rivera-Pérez W., Barquero-Calvo E., Chaves A. J. (2021). Effect of the use of probiotic *Bacillus subtilis* (QST 713) as a growth promoter in broilers: an alternative to bacitracin methylene disalicylate. *Poultry Science*.

[13] Tsukahara T., Kimura Y., Inoue R., Iwata T. (2020). Preliminary investigation of the use of dietary supplementation with probiotic *Bacillus subtilis strain* QST713 shows that it attenuates antimicrobial-induced dysbiosis in weaned piglets. *Animal Science Journal*.

[14] Rychen G., Aquilina G., Azimonti G. (2017). Safety and efficacy of *Bacillus subtilis* PB6 (*Bacillus subtilis* ATCC PTA-6737) as a feed additive for sows. *EFSA Journal*.

[15] Jeong J., Kim J., Lee S., Kim I. (2015). Evaluation of *Bacillus subtilis* and *Lactobacillus acidophilus* probiotic supplementation on reproductive performance and noxious gas emission in sows. *Annals of Animal Science*.

[16] Taras D., Vahjen W., Macha M., Simon O. (2006). Performance, diarrhea incidence, and occurrence of *Escherichia coli* virulence genes during long-term administration of a probiotic *Enterococcus faecium* strain to sows and piglets. *Journal of Animal Science*.

[17] Taras D., Vahjen W., Macha M., Simon O. (2005). Response of performance characteristics and fecal consistency to long-lasting dietary supplementation with the probiotic strain *Bacillus cereus* var. toyoi to sows and piglets. *Archives of Animal Nutrition*.

[18] Alexopoulos C., Georgoulakis I. E., Tzivara A., Kritas S. K., Siochu A., Kyriakis S. C. (2004). Field evaluation of the efficacy of a probiotic containing *Bacillus licheniformis* and *Bacillus subtilis* spores, on the health status and performance of sows and their litters. *Journal of Animal Physiology and Animal Nutrition*.

[19] Wang C., Wei S., Xu B. (2021). *Bacillus subtilis* and *Enterococcus faecium* co-fermented feed regulates lactating sow’s performance, immune status and gut microbiota. *Microbial Biotechnology*.

[20] Liu H., Wang S., Zhang D. (2020). Effects of dietary supplementation with Pediococcus acidilactici ZPA017 on reproductive performance, fecal microbial flora and serum indices in sows during late gestation and lactation. *Asian-Australasian Journal of Animal Sciences*.

[21] Nam N. H., Sukon P. (2021). Noninfectious risk factors for intrapartum stillbirth in a swine farm in the north of Vietnam. *Veterinary World*.

[22] Nam N. H., Sukon P. (2020). Risk factors associated with stillbirth of piglets born from oxytocin-assisted parturitions. *Veterinary World*.

[23] Baxter E. M., Jarvis S., Sherwood L. (2009). Indicators of piglet survival in an outdoor farrowing system. *Livestock Science*.

[24] Baxter E. M., Jarvis S., D’Eath R. B. (2008). Investigating the behavioural and physiological indicators of neonatal survival in pigs. *Theriogenology*.

[25] Nam N. H., Sukon P. (2021). Risk factors associated with dystocia in swine. *Veterinary World*.

[26] Nam N. H., Sukon P. (2020). Associated factors for farrowing duration in sows with nutural farrowing in intensive conditions. *World’s Veterinary Journal*.

[27] Kritas S. K., Marubashi T., Filioussis G. (2015). Reproductive performance of sows was improved by administration of a sporing bacillary probiotic (*Bacillus subtilis* C-3102). *Journal of Animal Science*.

[28] Gu X. L., Li H., Song Z. H., Ding Y., He X., Fan Z. (2019). Effects of isomaltooligosaccharide and *Bacillus* supplementation on sow performance, serum metabolites, and serum and placental oxidative status. *Animal Reproduction Science*.

[29] Quiniou N., Dagorn J., Gaudré D. (2002). Variation of piglets’ birth weight and consequences on subsequent performance. *Livestock Production Science*.

[30] Quesnel H., Brossard L., Valancogne A., Quiniou N. (2008). Influence of some sow characteristics on within-litter variation of piglet birth weight. *Animal*.

[31] Zindove T., Dzomba E., Kanengoni A., ChiMonyo M. (2014). Variation in individual piglet birth weights in a large white × landrace sow herd. *South African Journal of Animal Science*.

[32] van Dijk A. J., van Rens B., van der Lende T., Taverne M. (2005). Factors affecting duration of the expulsive stage of parturition and piglet birth intervals in sows with uncomplicated, spontaneous farrowings. *Theriogenology*.

[33] Gadde U., Oh S. T., Lee Y. S. (2017). The effects of direct-fed microbial supplementation, as an alternative to antibiotics, on growth performance, intestinal immune status, and epithelial barrier gene expression in broiler chickens. *Probiotics and Antimicrobial Proteins*.

[34] Molna A. K., Podmaniczky B., Podmaniczky R. B. (2011). Effect of different concentrations *of Bacillus subtilis* on growth performance, carcase quality, gut microflora and immune response of broiler chickens. *British Poultry Science*.

[35] Sikandar A., Zaneb H., Younus M. (1970). Growth performance, immune status and organ morphometry in broilers fed *Bacillus subtilis*-supplemented diet. *South African Journal of Animal Science*.

[36] Urdaci M. C., Pinchuk I., Ricca E., Henriques A. O., Cutting S. M. (2004). Antimicrobial activity of *Bacillus probiotics*. *Bacterial Spore Formers: Probiotics and Emerging Applications*.

[37] Teo A. Y. L., Tan H.-M. (2005). Inhibition of *Clostridium perfringens* by a novel strain of *Bacillus subtilis* isolated from the gastrointestinal tracts of healthy chickens. *Applied and Environmental Microbiology*.

